# Characterization of osteoarthritic human knees indicates potential sex differences

**DOI:** 10.1186/s13293-016-0080-z

**Published:** 2016-06-02

**Authors:** Qingfen Pan, Mary I. O’Connor, Richard D. Coutts, Sharon L. Hyzy, Rene Olivares-Navarrete, Zvi Schwartz, Barbara D. Boyan

**Affiliations:** Department of Mechanical Engineering, Georgia Institute of Technology, Atlanta, GA USA; Center for Musculoskeletal Care, Yale University School of Medicine, New Haven, CT USA; Department of Orthopaedics, University of California at San Diego, San Diego, CA USA; Department of Biomedical Engineering, Virginia Commonwealth University, Richmond, VA USA; Department of Periodontics, University of Texas Health Science Center at San Antonio, San Antonio, TX USA; Wallace H. Coulter Department of Biomedical Engineering, Georgia Institute of Technology, Atlanta, GA USA; School of Engineering, Virginia Commonwealth University, 601 West Main Street, Suite 331, Richmond, VA 23284 USA

**Keywords:** Osteoarthritis, Chondrocytes, Osteoblasts, Estrogen, Testosterone, Dihydrotestosterone, 1α,25(OH)_2_D_3_, 24R,25(OH)_2_D_3_, Vitamin D

## Abstract

**Background:**

The prevalence of osteoarthritis is higher in women than in men in every age group, and overall prevalence increases with advancing age. Sex-specific differences in the properties of osteoarthritic joint tissues may permit the development of sex-specific therapies. Sex hormones regulate cartilage and bone development and homeostasis in a sex-dependent manner. Recent in vitro studies show that the vitamin D_3_ metabolite 1α,25-dihydroxyvitamin D3 [1α,25(OH)_2_D_3_] also has sex-specific effects on musculoskeletal cells, suggesting that vitamin D_3_ metabolites may play a role in osteoarthritis-related sex-specific differences. The purpose of this study was to determine if sex-specific differences exist in synovial fluid and knee tissues isolated from male and female patients with severe knee osteoarthritis. We determined the presence of vitamin D_3_ metabolites, inflammatory cytokines, growth factors, and matrix metalloproteinases (MMPs) in synovial fluid and assessed responses of articular chondrocytes and subchondral osteoblasts to 17β-estradiol, dihydrotestosterone, and 1α,25(OH)_2_D_3_.

**Methods:**

Samples from knee joints of 10 Caucasian male and 10 Caucasian female patients with advanced osteoarthritis aged 65 to 75 years were obtained from total knee arthroplasty. Vitamin D metabolites, cytokines, MMPs, and growth factors in the synovial fluid were measured. Primary cultures of chondrocytes were isolated from fibrillated articular cartilage adjacent to osteoarthritis lesions and minimally affected cartilage distal to the lesion. Osteoblasts were isolated from the subchondral bone. Expression of receptors for 17β-estradiol and 1α,25(OH)_2_D_3_ was assessed by real-time PCR. Chondrocytes and osteoblasts were treated with 10^−8^ M 17β-estradiol, dihydrotestosterone, or 1α,25(OH)_2_D_3_ and effects on gene expression and protein synthesis determined.

**Results:**

Histology of the articular cartilage confirmed advanced osteoarthritis. Sex differences were found in synovial fluid levels of vitamin D metabolites, cytokines, and metalloproteinases as well as in the cellular expression of receptors for 17β-estradiol and 1α,25(OH)_2_D_3_. Male cells were more responsive to 1α,25(OH)_2_D_3_ and dihydrotestosterone, whereas 17β-estradiol-affected female cells.

**Conclusions:**

These results demonstrate that there are underlying sex differences in knee tissues affected by osteoarthritis. Our findings do not address osteoarthritis etiology but have implications for different prevention methods and treatments for men and women. Further research is needed to better understand these sex-based differences.

**Electronic supplementary material:**

The online version of this article (doi:10.1186/s13293-016-0080-z) contains supplementary material, which is available to authorized users.

## Background

Osteoarthritis (OA) is a leading cause of pain and disability in adults. Nearly one in two people may develop symptomatic knee OA by age 85 [1]. OA affects the whole joint, including the articular cartilage, subchondral bone, muscle, tendons, meniscus, and synovium, and impacts daily life and function. Symptoms may include joint pain, tenderness, stiffness, and locking [2]. A variety of causes, including hereditary, developmental, metabolic, and mechanical deficits, may initiate processes leading to cartilage loss. Risk factors for developing OA include patient sex, race/ethnicity, joint instability, obesity, joint trauma, and age. Epidemiologic studies show sex-specific differences in the prevalence and severity of OA [3, 4]. On average, the rate of arthritis is 58 % higher in women than in men [5]. Women report more pain due to OA than men report and are more likely to have reductions in function and quality of life [6]. Biomechanical differences between men and women may contribute to differences in OA prevalence, although data supporting this are not clear [7]; thus, other sex differences may be involved.

Sex hormones are important regulators of cartilage biology. Ovariectomized Cynomolgus monkeys exhibit histopathologic features typical of OA, suggesting that estrogen has a protective effect [8]. Studies using a mouse model of OA support this; ovariectomized female mice had more OA than intact mice, but orchidectomized male mice had less severe OA than intact males [9]. A number of studies have shown sexual dimorphism in the response of chondrocytes and osteoblasts to 17β-estradiol (E2) and dihydrotestosterone (DHT) at the cellular level [10–12]; however, the effects of sex hormones on the incidence and progression of OA of the knee, as well as on the regenerative potential of affected cartilage, are poorly understood.

Studies indicate that bone and cartilage cells exhibit sex-specific responses to the vitamin D metabolites 1α,25-dihydroxyvitamin D3 [1,25(OH)_2_D_3_] and 24R,25-dihydroxyvitamin D3 [24,25(OH)_2_D_3_] [13–15]. In addition to its role in maintaining calcium homeostasis and bone metabolism [16–18], recent evidence suggests that 1,25(OH)_2_D_3_ plays important roles in cartilage remodeling and participates in the inflammatory response [19, 20]. Epidemiologic studies have correlated low serum levels of 25-hydroxyvitamin D_3_ [25(OH)D_3_], the precursor of 1,25(OH)_2_D_3_ and 24,25(OH)_2_D_3_, with OA progression [21]. Moreover, vitamin D supplementation had a protective effect against OA in rats by reducing messenger RNAs (mRNAs) for matrix metalloproteinase (MMP)-3, interleukin-1β (IL1β), and tumor necrosis factor alpha (TNFα), factors associated with cartilage degradation and inflammation [22]. Interestingly, 1α,25(OH)_2_D_3_ induced production of E2 in female rat chondrocytes but not in male chondrocytes, further demonstrating that there is sexual dimorphism at the cellular level [23].

As a first step in understanding how sex differences might have an impact on OA, we investigated whether differences were present in patients with OA sufficiently severe to warrant total joint replacement. We reasoned that the disease was comparable in patients of both sexes, and while the etiology of the disease in any one patient was unknown, all patients would receive the same treatment. Thus, identified differences would be more likely to be intrinsic to the sex of the patient, rather than random non-specific observations. We examined serum and synovial fluid obtained at the time of surgery as well as the morphology of the articular cartilage. Also, we isolated chondrocytes and osteoblasts and examined their responses to 1α,25(OH)_2_D_3_, E2, and DHT.

## Methods

### Patient population

Surgeries and preclinical assessments were performed at Mayo Clinic, Jacksonville, FL, USA. Bench studies were conducted at Georgia Institute of Technology, Atlanta, GA, USA, and Virginia Commonwealth University, Richmond, VA, USA. Analyses for the studies were performed at all locations listed under affiliations. Each author certifies that his or her institution approved the human protocol for this investigation (Mayo Clinic - 11-001468; Georgia Tech - H11239; VCU - HM15270), and that all investigations were conducted in conformity with ethical principles of research, and that informed consent for participation in the study was obtained.

Twenty white, non-Hispanic patients (10 men/10 women) between the ages of 65 and 75 years, who were undergoing total knee arthroplasty (TKA) due to severe OA of the knee, were included in our study. Patients with inflammatory arthritis, osteonecrosis, prior upper tibial osteotomy, premenopausal women, patients aged less than 65 years or aged greater than 75 years; who were insulin-dependent or diabetic, with a BMI greater than 30 kg/m^2^, or history of knee infection were excluded from our study (Table 1). The study size of 20 was powered based on serum 25(OH)D_3_ levels, given an alpha of 0.05, a standard deviation of 17, an effect size of 22.5, and power of 0.8.

**Table 1:** Patient demographics and clinical history

Variable	Female	Male	*p* value^a^
Median age (IQR) - years	70 (68, 71)	69 (66, 72)	0.42
Median body mass index (IQR) - kg/m^2^	26.2 (25.6, 28.1)	27.0 (24.3, 28.6)	0.34
Vitamin D use - no. (%)^b^			0.14
Currently	6 (60 %)	3 (30 %)	
Previously	3 (30 %)	2 (20 %)	
Never	1 (10 %)	5 (50 %)	
Median 25-hydroxy D3 (IQR) - ng/ml	42.1 (33.7, 47.2)	38.4 (31.0, 52.6)	0.79
Bisphosphonates use - no. (%)^b^			0.47
Previously	2 (20 %)	0 (0 %)	
Never	8 (80 %)	10 (100 %)	
Estrogen use - no. (%)^b^			<0.001
Currently	4 (40 %)	0 (0 %)	
Previously	4 (40 %)	0 (0 %)	
Never	2 (20 %)	10 (100 %)	
Previously injured a knee so badly it was difficult to walk for at least a week - no. (%)	0 (0 %)	3 (30 %)	0.21
Prior knee surgery - no. (%)	6 (60 %)	6 (60 %)	1.00
Prior intra-articular injections of steroids - no. (%)	7 (70 %)	5 (50 %)	0.65
Median no. of steroid injections (IQR)	1 (0, 1)^c^	1 (0, 3)	0.58
Prior intra-articular injection of hyaluronic acid - no. (%)	3 (30 %)	5 (50 %)	0.65
Median no. of hyaluronic injections (IQR)	0 (0, 1)	2 (0, 3)	0.30
Knee to be replaced - no. (%)			1.00
Right	6 (60 %)	6 (60 %)	
Left	4 (40 %)	4 (40 %)	

*IQR* interquartile range

^a^ *p* values result from a Wilcoxon rank sum test for numerical variables and Fisher’s exact test for categorical variables

^b^ *p* values are based on categorization as currently or previously used vs. never used

^c^Information was not available for 1 patient

Information collected at the Mayo Clinic before surgery included patient demographics (age, sex, body mass index [BMI]) and medication and supplement use (vitamin D supplements, bisphosphonates, and estrogen). In addition, we used several rating scales to provide baseline information on the two patient cohorts in the event that sex differences were observed. These included the Short Form-12 (SF-12) Health Survey, the Western Ontario & McMaster Universities Arthritis Index (WOMAC), and the Physical Activity Scale for the Elderly (PASE) functional scale tests. Serum 25(OH)D_3_ levels and standard radiographs were obtained for each patient. Within 2 weeks of surgery, patients completed the Osteoarthritis Research Society International-Outcome Measures in Rheumatology (OARSI-OMERACT) pain scale [24], the knee pain map [25], and pressure–pain thresholds at the knee using JTECH Tracker wireless software (V.5; JTECH Medical, Midvale, UT, USA) [26, 27].

Although not statistically significant, there was some evidence to suggest that previous or current vitamin D use was more common in women patients than in men patients (90 vs. 50 %; *p* = 0.14). As expected, only the female cohort reported previous or current estrogen use (80 vs. 0 %; *p* < 0.001). There was no evidence of a difference in age, BMI, bisphosphonate use, prior knee surgery, prior intraarticular steroid injections, or prior intraarticular hyaluronic acid injections between men and women patients (*p* ≥ 0.21).

Per preoperative radiographic and knee pain assessments, the pain-pressure threshold 1 cm above the medial joint line was lower in women than men (median, 3.0 vs. 5.1 kg; *p* = 0.007) (Table 2). There was no evidence of a difference in patient serum levels of 25(OH)D_3_, radiographic assessment, knee pain, or areas of knee pain (*p* ≥ 0.37). There was no evidence of a difference between male and female patients in the pain and functional assessments before surgery, including the WOMAC, OARSI-OMERACT, and SF-12 (*p* ≥ 0.36) (Table 3).

**Table 2:** Preoperative knee pain and radiographic assessment

Variable	Female	Male	*p* value^a^
Radiographic assessment			
Kellgren-Lawrence Grading Scale - no. (%)			0.37
Grade 3	1 (10 %)	0 (0 %)	
Grade 4	9 (90 %)	10 (100 %)	
Median pain-pressure threshold (IQR) - kg			
Medial joint line	4.5 (3.7, 5.1)	5.3 (4.1, 6.9)	0.16
1 cm above the medial joint line	3.0 (2.7, 3.8)	5.1 (4.5, 6.2)	0.007
1 cm below the medial joint line	4.0 (3.5, 4.1)	5.6 (4.0, 6.8)	0.10
Area(s) of knee pain - no. (%)^b^			
Localized			
Superior lateral	0 (0 %)	1 (10 %)	1.00
Patella	0 (0 %)	1 (10 %)	1.00
Medial joint line	3 (30 %)	2 (20 %)	1.00
Inferior lateral	1 (10 %)	0 (0 %)	1.00
Inferior medial	1 (10 %)	0 (0 %)	1.00
Regional			
Medial	1 (10 %)	1 (10 %)	1.00
Patella	1 (10 %)	2 (20 %)	1.00
Diffuse pain	4 (40 %)	3 (30 %)	1.00

^a^ *p* values result from a Wilcoxon rank sum test for numerical variables and Fisher’s exact test for categorical variables

^b^More than one location per patient was possible. Pain was not reported at any of the following locations: superior medial, lateral joint line, back of knee, lateral (regional), and back of knee (regional)

**Table 3:** Pain and functional assessment prior to surgery

Variable	Female	Male	*p* value^a^
WOMAC			
Median total raw score (IQR)	39 (32, 47)	45 (27, 55)	0.76
Median pain score (IQR)	8.5 (7, 10)	9.0 (5, 12)	0.73
Median stiffness score (IQR)	4.0 (2, 5)	4.0 (3, 6)	0.64
Median difficulty performing daily activities score (IQR)	28 (21, 33)	32 (18, 38)	0.70
OARSI-OMERACT pain scale			
Median total pain score (IQR)	49 (30, 59)	42 (39, 61)	0.88
Median constant pain subscore (IQR)	45 (20, 55)	38 (25, 45)	0.36
Median intermittent pain subscore (IQR)	48 (42, 63)	50 (42, 67)	0.70
SF-12			
Median physical scale (IQR)	33 (29, 42)^b^	38 (30, 39)	0.55
Median mental scale (IQR)	61 (54, 63)^b^	57 (54, 66)	0.90

^a^ *p* values result from a Wilcoxon rank sum test

^b^Information was not available for 1 patient

**Fig. 1:**
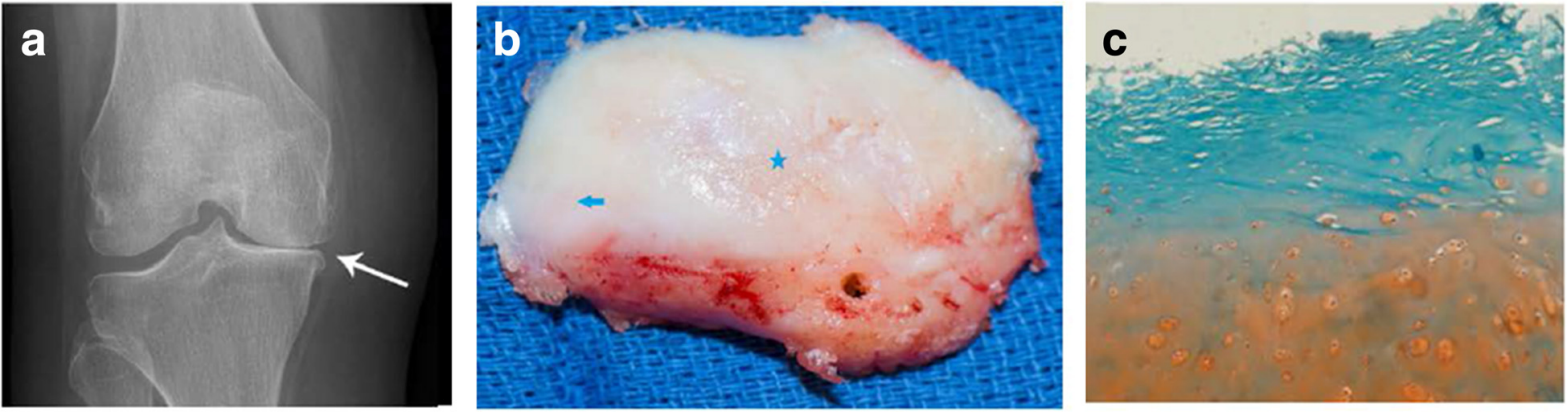
Gross changes in osteoarthritic joints. **a** Radiograph showing joint narrowing and bone-to-bone contact. **b** Articular cartilage showing areas of minimal fibrillation (*arrow*) and areas of maximum erosion (*star*). **c** Safranin-O-stained fibrillated cartilage showing loss of proteoglycan

All patients exhibited similar morphologic and histological features. All patients had radiographic evidence of joint narrowing and bone-bone contact (Fig. 1a).

### Sample collection

Fresh human tissue was obtained from consenting patients undergoing elective TKA at Mayo Clinic, Jacksonville, FL, USA. Synovial fluid was aspirated from the knee joint prior to skin incision, snap frozen, and stored at −80 °C. Bone, with overlaying cartilage specimens from the distal and posterior medial and lateral femoral condyles, and the proximal tibial bone cut were also obtained. Bone/cartilage specimens were shipped, on ice, to the Institute for Bioengineering and Bioscience at the Georgia Institute of Technology (Atlanta, GA, USA) or the School of Engineering, Institute for Engineering and Medicine at Virginia Commonwealth University (VCU; Richmond, VA, USA). Upon receipt, knee tissues were dissected, separating articular cartilage, subchondral bone, synovial membrane, and meniscus. Tissues were coded using de-identified patient numbers provided by Mayo Clinic. Articulating surfaces of the tibia and femur were photographed. Chondrocytes and osteoblasts were isolated at the time of receipt; meniscus, synovium, bone, and cartilage were processed for histology. Synovial fluid was thawed and aliquoted, then stored at −80 °C until used. All assays performed at VCU were blinded to donor sex.

### Histology

Following fixation in 10 % formalin, cartilage was decalcified (Decal Chemical Corporation, Tallman, NY, USA) for 16 h on a rotating platform before undergoing dehydration in a series of 95 and 100 % ethanol and xylene washes. Samples were embedded in paraffin; 7-μm sections were stained with safranin-O to assess glycosaminoglycan content.

### Synovial fluid

Synovial fluid levels of 1,25(OH)_2_D_3_ were measured according to manufacturer’s instructions using a human 1,25(OH)_2_D_3_ ELISA kit (DHVD_3_, Novatein Biosciences, Cambridge, MA, USA). Levels of 25(OH)D_3_ were measured according to manufacturer’s instructions using a human 25-hydroxyvitamin D_3_ ELISA kit (25HVD_3_, Novatein Biosciences). In addition, we used an ELISA kit from Immunodiagnostic Systems (Gaithersburg, MD, USA) that measured 25(OH)D_3_, 25(OH)D_2_, and 24,25(OH)_2_D_3_. We then approximated the amount of 24,25(OH)_2_D_3_ plus 25(OH)D_2_ by subtracting the values for 25(OH)D_3_ that had been obtained using the Novatein Biosciences assay. Because other vitamin D metabolites might have also been present, including 1,24,25(OH)_3_D_3_, mass spectrometry (Thermo Vantage Triple Quadrupole Mass Spectrometer; Biotrial, Quebec, Canada) was used to measure 24,25(OH)_2_D_3_ specifically. Data were calculated per milliliter of synovial fluid.

Inflammatory cytokines and MMPs present in the synovial fluid were measured using a Luminex screening assay (R&D Systems, Minneapolis, MN, USA). This assay uses superparamagnetic beads coated with analyte-specific antibodies. Transforming growth factor beta-1 (TGFβ1), TGFβ2, and TGFβ3 levels were measured using a magnetic bead-based multiplex assay (Bio-Rad, Hercules, CA, USA). Sulfated glycosaminoglycan levels were measured using the dimethyl methylene blue assay (Sigma-Aldrich, St. Louis, MO, USA) [28]. In all cases, data were determined per milliliter of synovial fluid.

### Chondrocyte cultures

Articular cartilage was harvested by sharp dissection from areas of minimal fibrillation and areas of maximum erosion (Fig. 1b). Cartilage pieces were minced and incubated in Dulbecco’s modified Eagle’s medium (DMEM; Thermo Fisher, Waltham, MA, USA) containing 0.25 % trypsin (Life Technologies, Carlsbad, CA, USA) for 30 min at 37 ° C. After discarding the trypsin digest, chondrocytes were isolated from cleaned cartilage fragments by incubating them for 16 h in 0.03 % type-II collagenase (Worthington Biosciences, Lakewood, NJ, USA) in DMEM containing 100 U/mL penicillin and 100 μg/mL streptomycin. Cells were separated from remaining cartilage pieces by straining the mixture through a 40-μm filter. Cells were cultured in DMEM supplemented with 10 % fetal bovine serum (Life Technologies) and 100 U/mL penicillin and 100 μg/mL streptomycin. A preliminary study demonstrated that first-passage cells retained their chondrocyte phenotype, whereas cells at later passages did not. Therefore, passage 1 cells were used for all experiments and cells were seeded at 15,000/cm^2^ for all experiments. We have successfully identified differences at the cellular level with as few as three donors per group [29], but we opted to culture the cells from all 20 patients. Unfortunately, there was not sufficient tissue available in some cases; in other instances, cell growth was inadequate. The final sample size for the cell culture studies was 6 males and 6 females.

In order to determine if chondrocytes were competent to respond to hormone treatment, mRNA levels for receptors for 1,25(OH)_2_D_3_ (vitamin D receptor [VDR] and protein disulfide isomerase A3 [PDIA3]) or E2 (ERα66 [ESR1] and ERα36) were measured in chondrocytes from both fibrillated and minimally affected OA tissues via real-time qPCR [30]. The cells were further characterized with respect to basal expression of inflammatory cytokines (IL1A, IL1B, IL6, IL8, and IL10) [31]. Related work has shown that differentiation of chondrocytes in response to 1,25(OH)_2_D_3_ involves signaling via wingless-Int pathway molecules (WNT3A, WNT5A, CTNNB, DKK1, and DKK2) [32]; therefore, we also measured RNA expression for these proteins. Primers for each of the mRNAs are shown in Additional file 1: Table S1.

RNA was harvested using a TRIzol (Invitrogen, Carlsbad, CA, USA) extraction method following manufacturer’s protocol. RNA was quantified using a NanoDrop spectrophotometer (Thermo Scientific, Waltham, MA, USA) and specific mRNAs (250 ng) were amplified using reverse transcription (High-Capacity cDNA Reverse Transcription Kit; Life Technologies). Starting quantities of mRNA were determined using SybrGreen chemistry (Power SYBR; Green PCR Master Mix, Life Technologies) in a StepOne Plus imaging system (Life Technologies). All gene expression was normalized to glyceraldehyde-3-phosphate dehydrogenase (GAPDH).

In order to examine if there were sex differences in response to hormonal treatment, confluent first passage chondrocytes were treated with 10^−8^ M 1α,25(OH)_2_D_3_ (Enzo Life Sciences, Plymouth, PA, USA) or 10^−8^ M E2 (Sigma-Aldrich, St. Louis, MO, USA). In addition, cells were treated with 10^−7^M 24R,25(OH)_2_D_3_ (Enzo Life Sciences) or 10^−8^ M 5α-DHT (Sigma-Aldrich). mRNAs for aggrecan (ACAN), type-II collagen (COL2A1), and cartilage oligomeric matrix protein (COMP) were measured at 12 h using real-time qPCR. Primers used are listed in Additional file 1: Table S1.

To measure alkaline phosphatase specific activity, confluent first-passage cells were treated for 24 h with either vehicle (0.01 % ethanol), or 10^−8^ M 1α,25(OH)_2_D_3_ or E2. Cells were harvested by trypsin digestion and were lysed in Triton X-100 (Sigma-Aldrich). Enzyme activity was measured by assaying the release of *p*-nitrophenol from *p*-nitrophenylphosphate at a pH of 10.2 in the cell lysates and was normalized to total protein content of the cell lysates (BCA Protein Assay; Thermo Fisher Scientific, Waltham, MA, USA) [33].

### Osteoblast cultures

Subchondral bone samples obtained from regions under minimally fibrillated cartilage and regions of maximal cartilage erosion were minced into small chips, incubated with 0.25 % trypsin for 30 min at 37 °C, and then washed with DMEM with 1 % penicillin-streptomycin three times. After washing, bone chips were cultured for 2 weeks in Petri dishes containing DMEM with 1 % penicillin-streptomycin and 10 % fetal bovine serum to enable osteoprogenitor cells to migrate out onto the dish surface. Media were changed once a week until cells reached confluence. Cells were then subcultured by seeding at 10,000 cells/cm^2^ for all experiments.

The presence of receptors for 1α,25(OH)_2_D_3_ and E2 was assessed as described for the chondrocyte cultures above. Response to the hormones was examined using confluent first-passage osteoblasts treated with either vehicle (0.01 % ethanol) or 10^−8^ M 1α,25(OH)_2_D_3_, E2 or DHT for 24 h. The conditioned media were collected, and levels of secreted osteocalcin and osteoprotegerin were determined. Osteocalcin was measured using a commercially available radioimmunoassay following manufacturer’s instructions (Biomedical Technologies, Stoughton, MA, USA) [34]. Osteoprotegerin was measured by ELISA (R&D Systems, Minneapolis, MN, USA) [35]. After decanting the conditioned media, cells were harvested by trypsin digestion and alkaline phosphatase-specific activity was determined in cell lysates. DNA was measured using a fluorometric assay from Promega (Madison, WI, USA). Levels of secreted factors in the conditioned media were normalized to total DNA.

### Statistical analysis

Clinical data were summarized for each sex by the sample median and interquartile (IQR) range for numerical variables whereas categorical variables were summarized by frequency and percentage. Comparisons between men and women were evaluated using a Wilcoxon rank sum test for numerical variables, Cochran-Armitage test for trend for ordered variables, and Fisher exact test for categoric variables. Statistical significance was considered at *p* < 0.05 without adjustment for multiple testing, owing to the exploratory and hypothesis-generating nature of the study. The considerable number of tests performed increased the chance of a type-I error, and interpretation of study results should consider this. Additionally, the small sample size increased the chance of a type-II error (i.e., false-negative association). Statistical analyses were performed using SAS statistical software (V. 9.3; SAS Institute Inc.; Cary, NC, USA).

Data from the cell studies are presented as the mean ± standard error (SEM) of six independent cultures for each patient per variable. Six patients of each sex were examined for the cell-hormone treatment study. Statistical significance was determined by ANOVA with post hoc Bonferroni modification of Student’s *t* test. For graphs labeled as treatment over control, the value of each sample from the treated group was divided by the mean of the control group. Each data point represents the mean ± SEM for six normalized values. The control is represented by a dashed line with a value equal to 1. Significance was determined by using the Mann-Whitney test. Significance was reached when *p* value was less than 0.05.

## Results

### Histology

Histology confirmed the radiographic diagnosis of severe OA. The articular cartilage was fibrillated and exhibited marked loss of sulfated glycosaminoglycans (sGAGs) (Fig. 1c).

### Synovial fluid

Synovial fluid composition differed between men and women patients (Table 4). Men had increased levels of MMPs 1, 7, 9, and 13. Hepatocyte growth factor (HGF), stem cell factor (SCF), and stem cell growth factor beta (SCGFβ) were all higher in male synovial fluid than in the synovial fluid of women patients. The amounts of sGAG, TGFβ1, and TGFβ2 were higher in men compared with women. Women had higher levels of inflammatory cytokines, IL2α, IL3, IL12p40, IL16, IL18, and TNFβ, and chondrocyte apoptosis-inducing factor (TRAIL) compared with men. Higher levels of macrophage stimulators (leukemia inhibitory factor [LIF], macrophage colony-stimulating factor [M-CSF], macrophage migration inhibitory factor [MIF]), and pro-inflammatory mediators (growth-regulated oncogene α [GRO-α], monocyte chemotactic protein-3 [MCP-3], and monokine induced by gamma interferon [MIG]) were found in women compared with men.

**Table 4:** Levels of inflammatory cytokines measured in synovial fluid

	Mean value (pg/ml) ± SEM			
	Male	Female	*p* value	
HGF	1075.00 ± 254.20	514.30 ± 46.10	0.0352	Males > females
IFN-α2	279.30 ± 50.09	159.80 ± 12.10	0.0207	
IL1α	389.00 ± 23.91	337.40 ± 9.80	0.0367	
MMP-1	2219.00 ± 539.60	1270.00 ± 84.74	0.049	
MMP-2	1981.00 ± 50.44	1811.00 ± 42.84	0.0248	
MMP-7	1189.00 ± 15.21	1147.00 ± 7.63	0.0173	
MMP-9	1565.00 ± 473.20	585.60 ± 135.50	0.0328	
MMP-12	39.20 ± 0.87	36.90 ± 0.46	0.0227	
MMP-13	441.90 ± 18.22	396.00 ± 7.241	0.0187	
SCF	478.00 ± 116.2	239.80 ± 21.45	0.0487	
SCGF-β	5503.00 ± 1713.20	1928.00 ± 433.60	0.0487	
TGFβ1	1723.00 ± 282.00	1070.00 ± 109.00	0.0464	
TGFβ2	35.79 ± 4.72	23.69 ± 2.74	0.0415	
sGAG	14.04 ± 2.02	6.25 ± 0.57	0.004	
GRO-α	459.40 ± 95.18	739.80 ± 61.36	0.0217	Females > males
IL2α	843.90 ± 166.50	1321.00 ± 120.20	0.0306	
IL3	806.90 ± 204.80	1289.00 ± 55.29	0.0395	
IL12p40	380.60 ± 81.14	578.20 ± 26.41	0.0342	
IL16	708.10 ± 148.50	1353.00 ± 247.00	0.0449	
LIF	309.60 ± 60.31	480.80 ± 41.60	0.0294	
MCP-3	147.70 ± 29.88	220.10 ± 17.42	0.0466	
M-CSF	748.80 ± 161.60	1156.00 ± 107.00	0.0468	
MIF	752.60 ± 172.70	1665.00 ± 357.50	0.0323	
MIG	1793.00 ± 321.80	4252.00 ± 1018.00	0.0371	
TNF-β	148.00 ± 42.95	252.20 ± 8.99	0.0304	
TRAIL	714.90 ± 156.20	1100.00 ± 79.97	0.0332	
β-NGF	5.67 ± 2.76	3.42 ± 0.83	0.4791	Not significant
CTACK	613.00 ± 112.90	925.30 ± 172.90	0.1621	
IL18	156.80 ± 8.56	160.90 ± 7.90	0.727	
IL1β	214.70 ± 99.25	128.80 ± 13.42	0.4113	
MMP-3	2898.00 ± 81.25	2961.00 ± 100.4	0.6544	
MMP-8	1183.00 ± 310.20	985.50 ± 103.40	0.4934	
SDF-1α	506.50 ± 79.08	461.70 ± 51.30	0.6259	

**Fig. 2:**
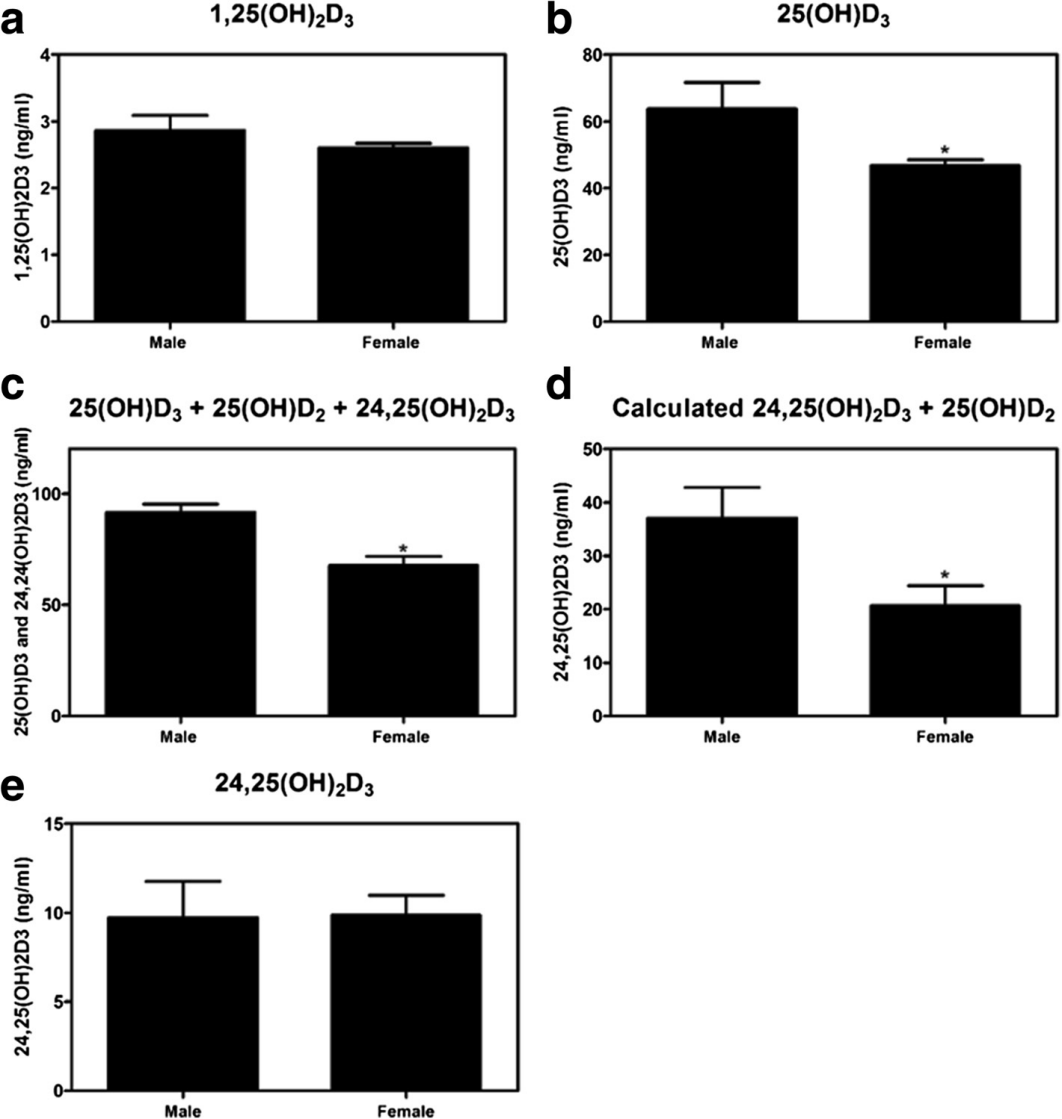
Vitamin D metabolites in synovial fluid. **a**–**d** Levels of vitamin D metabolites in the synovial fluid of 10 males and 10 females undergoing total joint arthroplasty were measured using ELISA and (**e**) mass spectrometry. ^*^ *p* < 0.05 compared with male

No sex-specific differences in the concentration of 1,25(OH)_2_D_3_ in synovial fluid were detected (Fig. 2a). Compared to men, women had less 25(OH)D_3_; 25(OH)D_3_ + 25(OH)D_2_ + 24,25(OH)_2_D_3_; and calculated 24,25(OH)_2_D_3_ + 25(OH)D_2_ in the synovial fluid (Fig. 2b–d); however, mass spectroscopy detected no differences in the levels of 24,25(OH)_2_D_3_ between women and men (Fig. 2e).

### Chondrocyte cultures

**Fig. 3:**
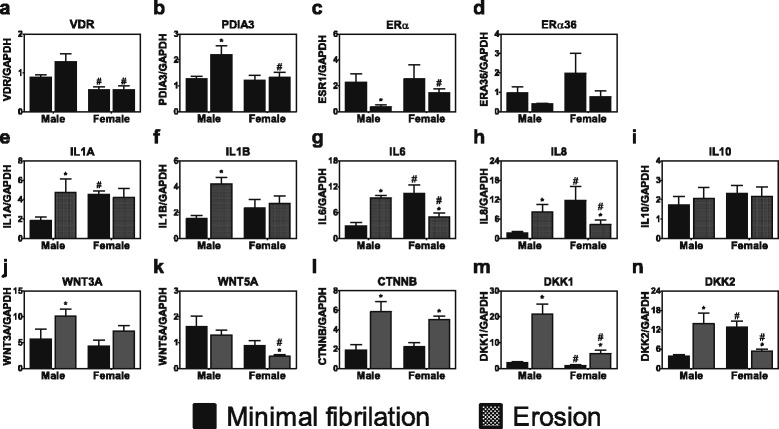
Phenotypic characteristics of female and male primary chondrocytes isolated from osteoarthritic knees. Primary chondrocytes were isolated from minimally fibrillated or eroded cartilage surfaces. mRNAs for 1α,25(OH)_2_D_3_ (VDR (**a**) and PDIA3 (**b**)); estrogen receptors (ERα66 (**c**) and ERα36 (**d**)); interleukins (IL1A (**e**), IL1B (**f**), IL6 (**g**), IL8 (**h**), and IL10 (**i**)); and WNT signaling molecules (WNT3A (**j**), WNT5A (**k**), β-catenin [CTNNB] (**l**), DKK1 (**m**), and DKK2 (**n**)) were measured using real-time qPCR. Data are the mean levels from 6 males and 6 females. ^*^ *p* < 0.05 compared with minimal fibrillation; ^†^ *p* < 0.05 compared with male

Chondrocytes expressed mRNAs for both receptors for 1,25(OH)_2_D_3_ (nVDR and PDIA3) and for ERα66 (ESR1) and ERα36 (Fig. 3a–d, respectively). Female chondrocytes from minimally fibrillated cartilage had less VDR mRNA than male cells but comparable levels of mRNAs for the other three receptors. Female cells isolated from eroded cartilage had less nVDR mRNA, less PDIA3 mRNA, and more ESR1 mRNA than male cells.

mRNAs for inflammatory cytokines were elevated in male cells from eroded cartilage compared to cells from minimally fibrillated cartilage (Fig. 3e–h). In contrast, female cells exhibited no differences in IL1A or IL1B mRNAs and expression of IL6 and IL8 was reduced in the female erosion cells compared with the minimally fibrillated cells. Female cells from minimally fibrillated cartilage had higher expression of IL1A, IL6, and IL8 than male cells from similar cartilage. No difference in expression of the anti-inflammatory cytokine IL10 was evident between male and female cells or as a function of the type of cartilage from which the cells were isolated (Fig. 3i).

Sex-specific differences in expression of WNT signaling molecules were observed, particularly in chondrocytes isolated from erosion cartilage. WNT3A, CTNNB, DKK2, and DKK2 were all expressed to a greater extent in male erosion cells than in cells from minimally eroded tissues (Fig. 3j, l–n). This was also the case for CTNNB and DKK1 in cells from female erosion tissue compared with minimally fibrillated female cells (Fig. 3l, m). However, WNT5A (Fig. 3k) and DKK2 (Fig. 3n) were reduced in female erosion tissue compared with minimally fibrillated tissue, and the levels of these two mRNAs were significantly lower than seen in male cells from similar tissues.

**Fig. 4:**
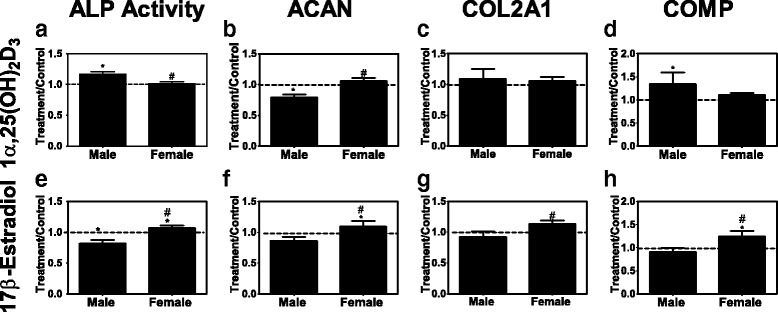
Phenotypic characteristics of female and male chondrocytes isolated from osteoarthritic knees. **a**–**d** Passage 1 chondrocytes were treated with 10^−8^ M 1α,25(OH)_2_D_3_ or 10^−8^ M E2. **e**–**h** Alkaline phosphatase specific activity (**a**, **e**) was measured in whole cell lysates. mRNAs for chondrocyte genes aggrecan (**b**, **f**), type-II collagen (**c**, **g**), and cartilage oligomatric matrix protein (**d**, **h**) were measured using real-time qPCR. Data show treatment versus vehicle control ratios of 6 male and 6 female donors. The *dashed line* represents the vehicle control (*dashed line* = 1). ^*^ *p* < 0.05 vs. control; ^†^ *p* < 0.05 vs. male

Chondrocytes responded to stimulation by vitamin D metabolites in a sex-specific manner. Male cells exhibited a more robust increase in alkaline phosphatase activity in response to 1α,25(OH)_2_D_3_ than female cells (Fig. 4a), but expression of ACAN was reduced (Fig. 4b). No sex-specific differences in expression of COL2A1 or COMP were observed (Fig. 4c, d). 24R,25(OH)_2_D_3_ inhibited alkaline phosphatase activity in female chondrocyte cultures compared with control cells and compared with male cells (Additional file 2: Figure S1A). 24R,25(OH)_2_D_3_ had a stimulatory effect on male cells compared with control cultures and to female cells treated with the vitamin D metabolite (Additional file 2: Figure S1B). 24R,25(OH)_2_D_3_ affected male and female cells comparably with respect to COL2A1 (increase) or COMP (decrease) (Additional file 2: Figure S1C, D).

E2 reduced alkaline phosphatase activity in male chondrocytes and stimulated it in female cells (Fig. 4e). E2 had no effect on expression of ACAN, COL2A1, or COMP in male cells, but it increased expression of all three mRNAs in female cells (Fig. 4f–h). DHT reduced alkaline phosphatase activity in male cells but had no effect on the enzyme in female cells (Additional file 2: Figure S1E). DHT had no effect on expression of ACAN, COL2A1, or COMP in cells of either sex (Additional file 2: Figure S1F–H).

### Osteoblast cultures

**Fig. 5:**
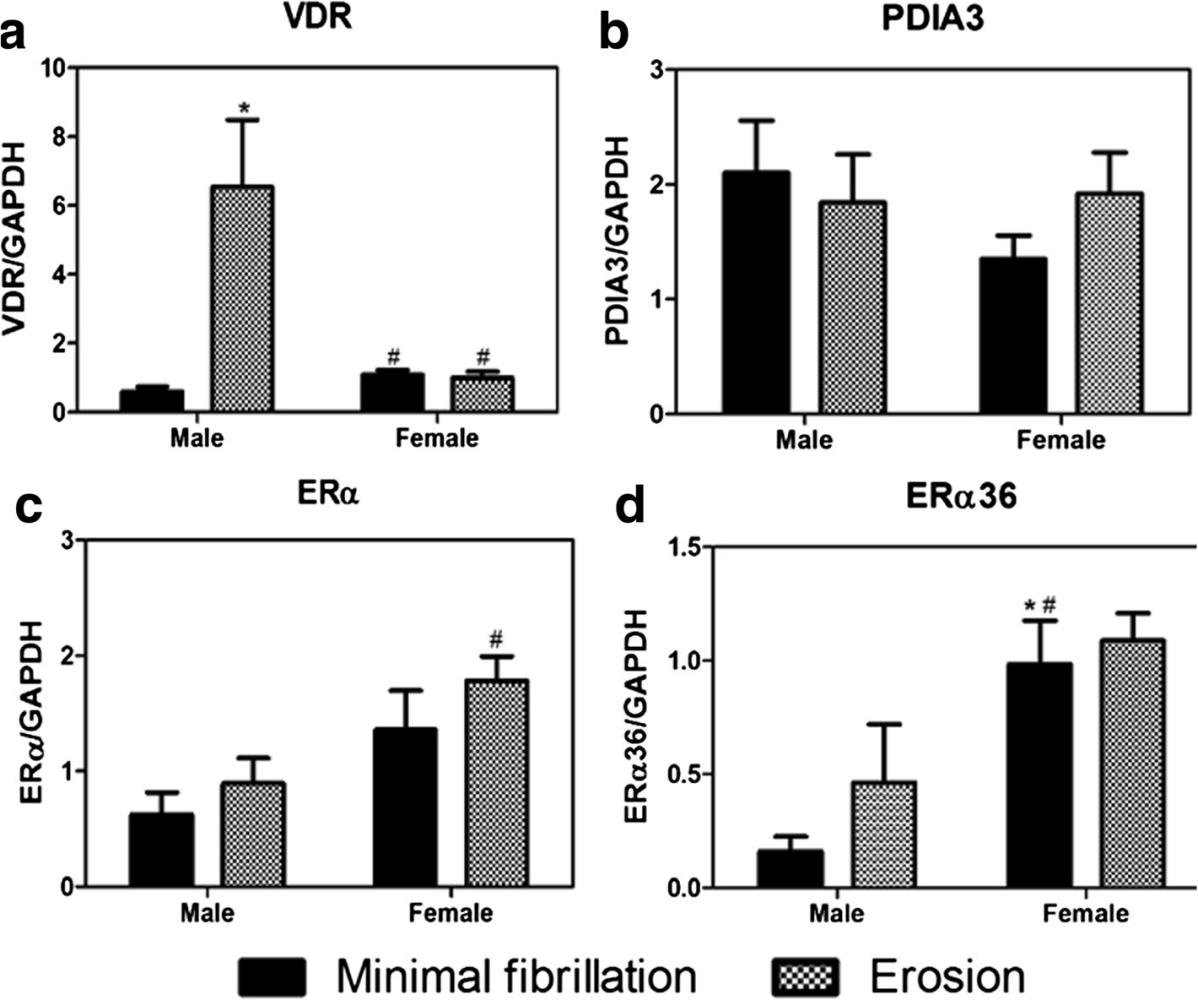
Phenotypic characteristics of female and male primary osteoblasts isolated from osteoarthritic knees. Female and male primary osteoblasts were isolated from subchondral bone. mRNAs for 1α,25(OH)_2_D_3_ (VDR (**a**) and PDIA3 (**b**)) and estrogen receptors ERα66 (ERα (**c**)) and ERα36 (**d**) were measured using real-time qPCR. Data are the mean ± SEM of 6 males and 6 females. ^*^ *p* < 0.05 vs. minimal fibrillation; ^†^ *p* < 0.05 vs. male

Male osteoblasts from erosion tissue expressed high levels of nVDR compared with minimally fibrillated male cells or with female cells from either site (Fig. 5a). No differences in expression of PDIA3 were observed (Fig. 5b). Female erosion cells expressed more ESR1 (ERα) and female minimally fibrillated osteoblasts expressed more ERα36 than male cells (Fig. 5c, d); however, no differences in expression of either E2 receptor were seen as a function of the tissue source for either males or females.

**Fig. 6:**
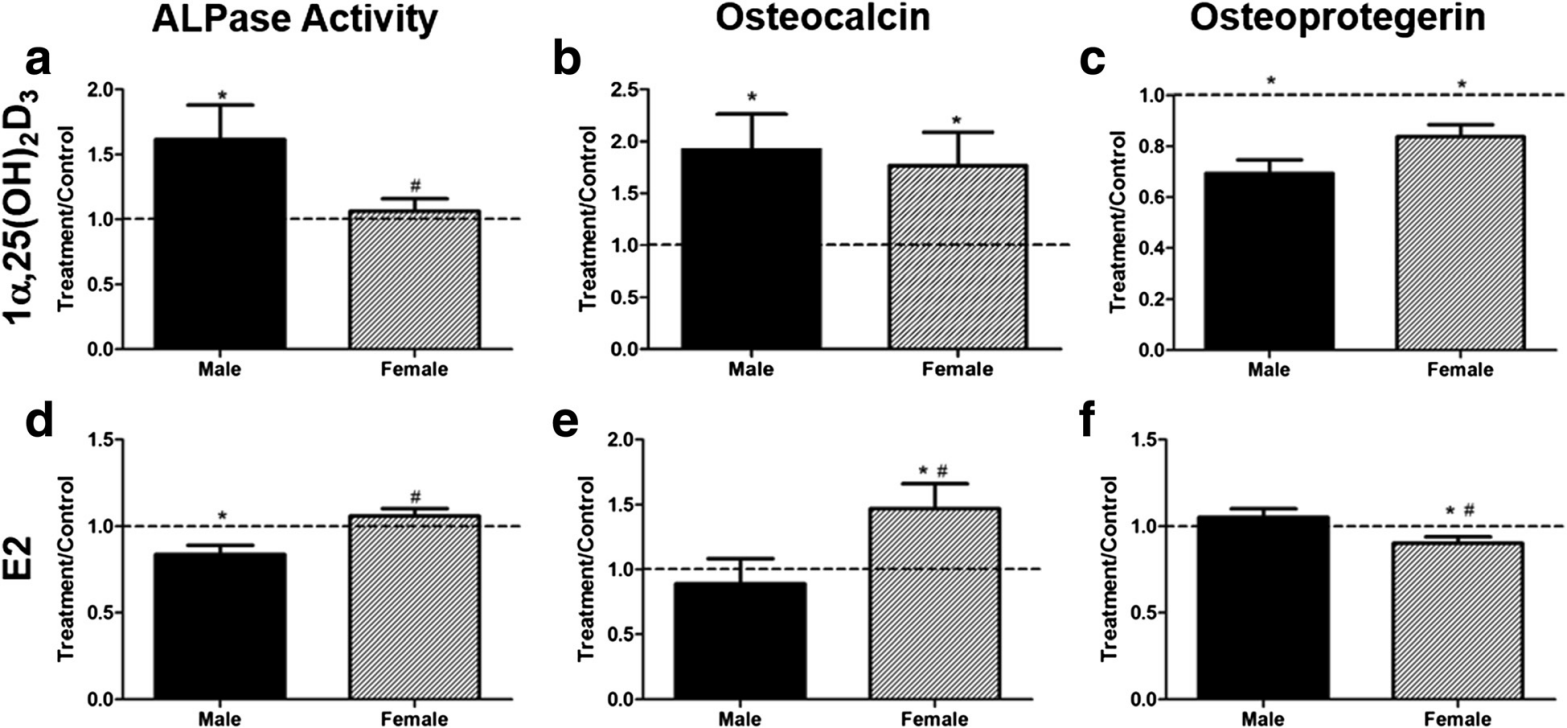
Phenotypic characteristics of female and male primary osteoblasts isolated from osteoarthritic knees. First-passage osteoblasts were treated with 10^−8^ M 1α,25(OH)_2_D_3_ (**a**–**c**) or 10^−8^ M E2 (**d**–**f**). Alkaline phosphatase-specific activity was measured in whole cell lysates (**a**, **d**). Protein levels of osteocalcin (**b**, **e**) and osteoprotegerin (**c**, **f**) were measured in conditioned media. Data show treatment compared with vehicle control ratios of the responses of 6 male and 6 female patients. The *dashed line* represents the vehicle control (*dashed line* = 1). ^*^ *p* < 0.05 vs. control; ^†^ *p* < 0.05 vs. male

In male osteoblasts, 1α,25(OH)_2_D_3_, increased alkaline phosphatase activity (Fig. 6a) and osteocalcin production (Fig. 6b) but decreased levels of osteoprotegerin (Fig. 6c). 1α,25(OH)_2_D_3_ had no effect on alkaline phosphatase activity in female cells, but its stimulatory effect on osteocalcin and inhibitory effect on osteoprotegerin were comparable to that seen in male cells. E2 inhibited alkaline phosphatase activity in male cells (Fig. 6d). It had no effect on enzyme activity in female osteoblast, but it stimulated osteocalcin production in the female cells (Fig. 6e), and it inhibited osteoprotegerin production (Fig. 6f). In contrast, DHT increased alkaline phosphatase activity in male osteoblasts (Additional file 3: Figure S2A), increased osteocalcin production (Additional file 3: Figure S2B), and reduced osteoprotegerin production (Additional file 3: Figure S2C). Female cells exhibited increased osteoprotegerin production in response to DHT.

## Discussion

Rather than investigating an underlying sex-dependent mechanism in the etiology of OA, the goal of this study was to screen a broad range of variables in tissues and fluids obtained during TKA to investigate whether there are sex differences in knee tissues affected by OA. The severity of OA in men and women study patients was comparable. Thus, detected differences could be ascribed to underlying sex-dependent traits rather than differences in disease status. Our results demonstrated that sex differences are present in OA-affected tissues. Not only were there differences in the composition of synovial fluid between the sexes, but chondrocytes and osteoblasts isolated from OA-affected cartilage and bone differed in a sex-dependent manner with respect to expression of hormone receptors and responded differentially to 1α,25(OH)_2_D_3_, 24R,25(OH)_2_D_3_, E2, and DHT. Moreover, articular chondrocytes from men and women patients exhibited sex differences in their expression of genes for inflammatory cytokines, enzymes involved in matrix degradation, and WNT signaling molecules involved in regulation of proliferation and differentiation.

Our patient population exhibited few clinical differences between men and women. There was no evidence of a difference in serum 25(OH)D_3_ levels, radiographic assessment, knee pain, or areas of knee pain prior to surgery between women and men. This could be due to the advanced stage of disease in our study population. Studies of younger patients have shown sex differences in knees with OA, with articular cartilage surface areas being 17.5 % to 23.5 % lower in women than in men [36], which supports the idea that sex differences contribute to disease progression, but once the advanced disease is established, sex differences are less important.

We did observe some clinical differences in the study patient population that were sex dependent. The pain-pressure threshold 1 cm above the medial joint line was lower in women than in men; however, the clinical significance of this is unclear. We did not test the pain-pressure threshold of anatomic regions outside the medial knee, and further study is warranted. We also found that women were more likely to have used or were using vitamin D and estrogen supplements compared with men. Studies have linked estrogen supplementation with reduced risk of OA, which could explain how a drop in estrogen in postmenopausal women could contribute to the severity of OA found in women [8, 37, 38] compared with males. Together, these data suggest that future studies should include younger postmenopausal women and age-matched men to better assess sex differences in their disease severity.

The synovial fluid composition was affected by the genetic sex of the patient in a number of ways. Men had higher levels of MMPs, the primary enzymes responsible for the degradation of cartilage, and HGF, which is synthesized by osteoblasts from the subchondral bone plate and produced at a higher rate by OA osteoblasts [39]. However, men had higher levels of growth factors, including TGFβs, SCF (stem cell factor), and SCGFβ (stem cell growth factor beta), and sGAGs compared with females, which have been shown to protect articular cartilage from degradation [40–42]. These results suggest that men may be more resistant to OA; although men had higher levels of matrix degradation enzymes, they also had more protective and growth-inducing factors that could aid in the repair and remodeling processes. Women had higher levels of inflammatory cytokines, especially IL18, which induces the production of PGE2 [43]. This is corroborated by studies showing that women experience more pain [6]. Women had less protective factors, which could explain their increased disease severity as they lose the protective effect of estrogen after menopause.

Vitamin D metabolites play important roles in skeletal development [44] and in maintaining cartilage and bone homeostasis [45, 46]. The ELISA kit used to measure 1,25(OH)_2_D_3_, a metabolite of 25(OH)D_3_ associated with Ca++ homeostasis as well as terminal differentiation of chondrocytes and osteoblasts [47–49], did not detect a difference in the synovial fluid levels between men and women. Because a suitable kit does not exist for measuring 24,25(OH)_2_D_3_, a metabolite associated with chondrocyte proliferation and resistance to apoptotic stimuli [50], we compared results using two kits, one that measured 25(OH)D_3_ alone and one that measured 25(OH)D_3_, 25(OH)D_2_, and 24,25(OH)_2_D_3_. Both kits identified reduced vitamin D metabolites in female synovial fluid, suggesting a possible reduction in 24,25(OH)_2_D_3_. However, when 24,25(OH)_2_D_3_ was measured directly by a commercial lab using mass spectroscopy, no sex difference was found. None of these methods is definitive, but taken together, they show a clear and reproducible finding that synovial vitamin D metabolites are regulated in a sex-dependent manner.

Studies using growth plate chondrocytes and healthy human articular chondrocytes have shown that the numbers of receptors vary in a sex-specific manner [11, 29, 51]. Our results examining gene expression also demonstrated sex-dependent differences. Chondrocytes from male erosion tissue and minimally fibrillated tissue expressed higher levels of mRNAs for the nuclear VDR whereas receptors for ERα66 (ESR1) were lower in cells from male erosion tissue than from female erosion tissue. Osteoblasts from male erosion tissue subchondral bone exhibited higher nuclear VDR expression, and female osteoblasts had higher ESR1 than male cells. We also found that male chondrocytes and osteoblasts were more responsive to 1α,25(OH)_2_D_3_ treatment than female cells. Previously, studies have shown that male osteoblasts have a more robust response to 1α,25(OH)_2_D_3_ [52], which supports our findings. Female cells were more responsive to estrogen treatment compared with male cells, which is supported by their greater expression of estrogen receptor mRNAs. The study results are supported by our previous observations using articular chondrocytes from young healthy men without OA, which showed that male cells had no response to E2 whereas female chondrocytes exhibited a marked response to E2 [29]. Studies have shown that chondrogenic progenitor cells respond differently to estrogen and testosterone in repairing OA cartilage, suggesting sexual dimorphism exists in cell-repair mechanisms in OA [53]. Collectively, our data suggest that the difference in the levels of VDR and PDIA3 and local and circulating 25(OH)D_3_ between men and women, could contribute to the more prevalent and severe cases of OA found in women.

Chondrocytes isolated from both erosion cartilage and minimally fibrillated cartilage exhibited sex-specific phenotypic traits. In addition to differences in receptor expression, there were a number of differences with respect to expression of mRNAs for pro-inflammatory cytokines. The level of these cytokines was comparable for eroded cartilage cells regardless of sex; however, in minimally fibrillated cartilage cells, women had higher levels of pro-inflammatory cytokines compared with men. This suggests that female chondrocytes may produce higher levels of inflammatory cytokines at an earlier stage of OA. Interestingly, the anti-inflammatory cytokine IL10 was expressed at comparable levels in cells from men and women irrespective of tissue source.

Expression of mRNAs for WNT family genes varied in a sex-specific manner as well, but the interpretation of these data will require further experimentation. Higher levels of WNT antagonists are suggested to inhibit chondrocyte hypertrophy. DKK1 inhibits MMP-13 and ADAMTS-4 expression in chondrocytes in response to WNT3a treatment [54]. Activation of WNT/β-catenin signaling in primary human chondrocytes inhibits basal IL1β-stimulated increases in MMP-1, -3, and -13 levels, possibly through inhibition of NFκB signaling [55, 56]. Men had higher levels of WNT signaling molecules, which is consistent with our findings that although men had higher levels of MMPs, they may be more resistant to OA as they also had more factors that are protective.

The underlying etiology of OA is not well understood. It has been hypothesized that mechanical instabilities lead to changes in the biomechanical properties of the articular cartilage and, ultimately, in the biochemical properties of the cells and extracellular matrix [7]. Recent data indicate that there are sex-specific differences at the molecular and cellular level that may exacerbate the effects of altered mechanical load [57–60]. Our data do not address disease etiology. While they do support the hypothesis that sex differences exist, all patients had advanced OA. Thus, it is not possible to attribute specific differences to a clinical outcome. It is possible, however, that sex differences may have contributed to differences in disease progression that led to the need for TKA.

This study had a number of limitations. We used passage 1 cells in our study to investigate the effect of hormone treatments on the cells. Human chondrocytes lose phenotypic traits when subpassaged, so it is preferable to use primary cells. Unfortunately, the number of primary cells was limited, necessitating the need for culture expansion. In a series of preliminary studies, we found that the first-passage cells expressed similar levels of chondrocyte and osteoblast markers to primary cells (data not shown). One of the limitations of our studies is that we could not get tissues from healthy human knees as a control; however, we addressed the limitation by using cartilage and subchondral bone from areas that showed minimal articular fibrillation.

## Conclusions

In this study, we identified sex-specific differences in synovial fluid, chondrocytes, and osteoblasts in advanced human knee OA tissues. Our understanding of such sex-specific differences is still very early, and additional research is needed. Understanding such sex differences in knee OA may allow for the introduction of sex-specific therapies and new treatment avenues for patients experiencing this often disabling condition.

## Additional files

Additional file 1: Table S1. Primers for real-time PCR. (DOCX 16 kb) Additional file 2: Figure S1. Phenotypic characteristics of female and male chondrocytes isolated from osteoarthritic knees. First-passage chondrocytes were treated with 10^−7^ M 24R,25(OH)_2_D_3_ (A-D) or 10^-8^ M DHT (E–H). Alkaline phosphatase-specific activity (A, D) was measured in whole cell lysates. mRNAs for chondrocyte genes aggrecan (B, F), type-II collagen (C, G), and cartilage oligomeric matrix protein (D, H) were measured using real-time qPCR. Data show treatment compared with vehicle control ratios of the responses of 6 male and 6 female patients. The dashed line represents the vehicle control (dashed line = 1). ^*^ *p* < 0.05 vs. control; ^†^ *p* < 0.05 vs. male. (TIF 1363 kb) Additional file 3: Figure S2. Phenotypic characteristics of female and male primary osteoblasts isolated from osteoarthritic knees. First-passage osteoblasts were treated with 10^−8^ M DHT. Alkaline phosphatase-specific activity (A) was measured in whole cell lysates. Protein levels of osteocalcin (B) and osteoprotegerin (C) were measured in conditioned media. Data show treatment compared with vehicle control ratios of the responses of 6 male and 6 female patients. The dashed line represents the vehicle control (dashed line = 1). ^*^ *p* < 0.05 vs. control; ^†^ *p* < 0.05 vs. male. (TIF 1003 kb)

## Abbreviations

sGAG: sulfated glycosaminoglycan
E2: 17β-estradiol
DHT: dihydrotestosterone
1α,25(OH)_2_D_3_: 1-alpha,25-dihydroxyvitamin D3
24R,25(OH)_2_D_3_: 24R,25-dihydroxyvitamin D3
VDR: canonical vitamin D receptor
PDIA3: protein disulfide isomerase family A member 3
ERα66: canonical estrogen receptor a
ERα36: shorter variant estrogen receptor
OA: osteoarthritis
HGF: hepatocyte growth factor
SCF: stem cell factor
SCGFβ: stem cell growth factor beta
TGFβ: transforming growth factor beta
TNFα and TNFβ: tumor necrosis factor alpha and beta
MIF: macrophage migration inhibitory factor
WNT: wingless-related integrated site a family of signaling molecules
DKK1: Dickkopf-1
IL1α etc.: interleukin-1 alpha
ACAN: mRNA for aggrecan
COL2A1: mRNA for type-II collagen alpha 1 chain
COMP: mRNA for cartilage oligomeric matrix protein
MMP: matrix metalloproteinase
TRAIL: TNF-related apoptosis-inducing ligand
LIF: leukemia inhibitory factor
M-CSF: macrophage colony-stimulating factor
GRO-α: growth-regulated oncogene α
MCP-3: monocyte chemotactic protein-3
MIG: monokine induced by gamma interferon
CTNNB: mRNA for beta-catenin
TKA: total knee arthroplasty
SF-12: Short Form-12 Health Survey
WOMAC: Western Ontario & McMaster Universities Arthritis Index
PASE: Physical Activity Scale for the Elderly
OARSI-OMERACT: Osteoarthritis Research Society International-Outcome Measures in Rheumatology pain scale
