# Supplementary material for: Characterization of osteoarthritic human knees indicates potential sex differences
Source: Biol Sex Differ. 2016 Jun 2;7:27. doi: 10.1186/s13293-016-0080-z (PMC4890516; doi:10.1186/s13293-016-0080-z)
Supplement: Additional file 1: Table S1. — Primers for real-time PCR. (DOCX 16 kb) [file 13293_2016_80_MOESM1_ESM.docx]

| ACAN  **Additional file 1: Table S1. Primers for Real-Time PCR** | F | TCA GCG GTT CCT TCT CCA G |
| --- | --- | --- |
|  | R | GCA GTT GTC TCC TCT TCT ACG |
| COL2A1 | F | QuantiTect Primer Assay |
|  | R |  |
| COMP | F | CCT GCG TTC TTC TGC TCA C |
|  | R | GCG TCA CAC TCC ATC ACC |
| CTNNB | F | GGC AGC AAC AGT CTT ACC |
|  | R | TCC ACA TCC TCT TCC TCA |
| DKK1 | F | CCA GAC CAT TGA CAA CTA CC |
|  | R | CAG GCG AGA CAG ATT TGC |
| DKK2 | F | TGA CTT GGG ATG GCA GAA TC |
|  | R | CAG AAA TGA CGA GCA CAG C |
| ESR1 | F | QuantiTect Primer Assay for ERα66 |
|  | R |  |
| ERα36 | F | GTGGTTTCCTCGTGTCTAAAGC |
|  | R | GGTGTTGAGTGTTGGTTGCC |
| IL1A | F | TCC CGG GGC TTG CAC ACA CCT T |
|  | R | ACT CTC CAC CCT GGC CCT GTT ACA |
| IL1B | F | TGG CAG AAA GGG AAC AGA AAG G |
|  | R | AAC AAA AGG GCT GGG GAT TGG |
| IL6 | F | CCT CGA GCC CAC CGG GAA CGA AA |
|  | R | GGG GTA CTG GGG CAG GGA AGG C |
| IL7 | F | TCT TCT TCT GTG CTG GAG ATG |
|  | R | GGA CCT TGT TAT GCT GTT GC |
| IL8 | F | GAC ATA CTC CCA AAC CTT TCC AC |
|  | R | AAA CCT CTC CAC AAC CCT CTG |
| IL10 | F | GGC TGA GGC TAC GGC GCT GTC A |
|  | R | CTTCACCTGCTCCACGGCCTTGC |
| PDIA3 | F | AAGAAGAAGGCACAGGAG |
|  | R | ATGAACTTCAGGGTCAGC |
| VDR | F | CAT CAG AAG GAG AAG GAA GG |
|  | R | TGA GGC AAC AGC ATT ATC C |
| WNT3A | F | CTGTAGCGAGGACATCGAGTTT |
|  | R | GGCACCTTGAAGTAGGTGTAG |
| WNT5A | F | TCT CAG CCC AAG CAA CAA GG |
|  | R | GCC AGC ATC ACA TCA CAA CAC |
