# Supplementary figures and images for: Characterization of osteoarthritic human knees indicates potential sex differences
Source: Biol Sex Differ. 2016 Jun 2;7:27. doi: 10.1186/s13293-016-0080-z (PMC4890516; doi:10.1186/s13293-016-0080-z)

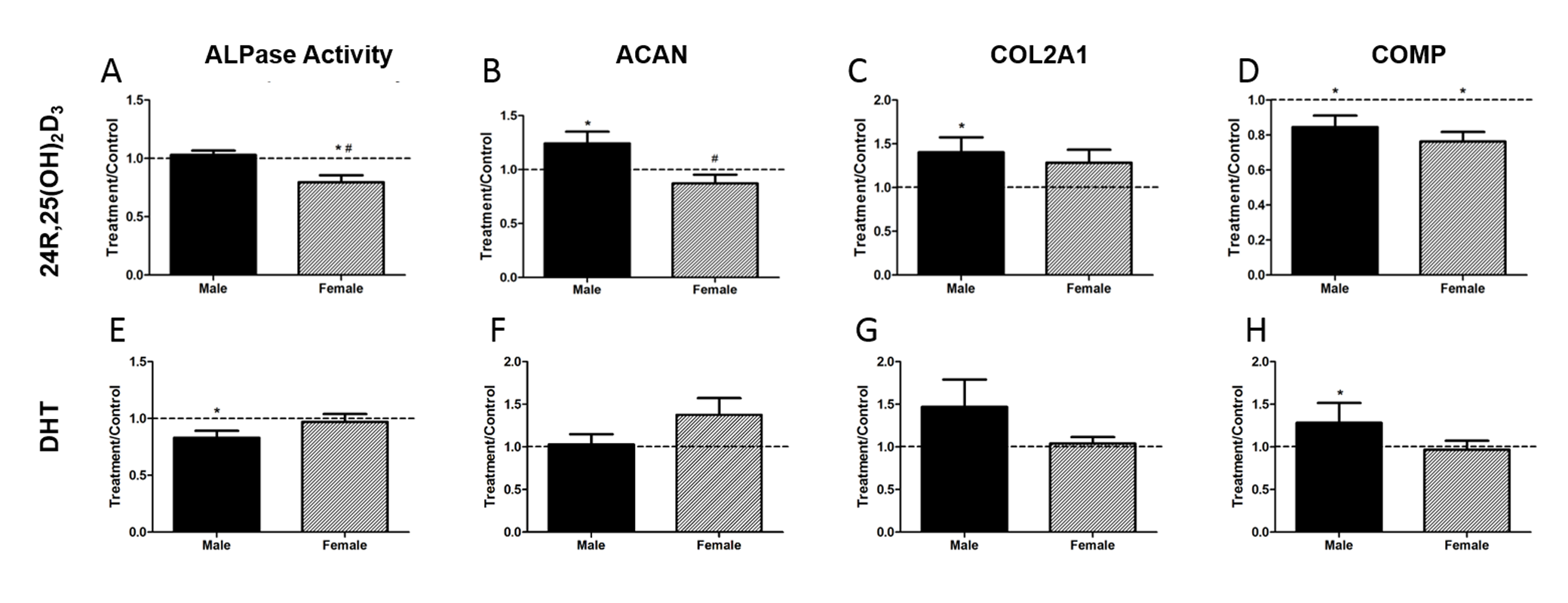

Supplement: Additional file 2: Figure S1. — Phenotypic characteristics of female and male chondrocytes isolated from osteoarthritic knees. First-passage chondrocytes were treated with 10−7 M 24R,25(OH)2D3 (A-D) or 10-8 M DHT (E–H). Alkaline phosphatase-specific activity (A, D) was measured in whole cell lysates. mRNAs for chondrocyte genes aggrecan (B, F), type-II collagen (C, G), and cartilage oligomeric matrix protein (D, H) were measured using real-time qPCR. Data show treatment compared with vehicle control ratios of the responses of 6 male and 6 female patients. The dashed line represents the vehicle control (dashed line = 1). * p < 0.05 vs. control; † p < 0.05 vs. male. (TIF 1363 kb) [file 13293_2016_80_MOESM2_ESM.tif]

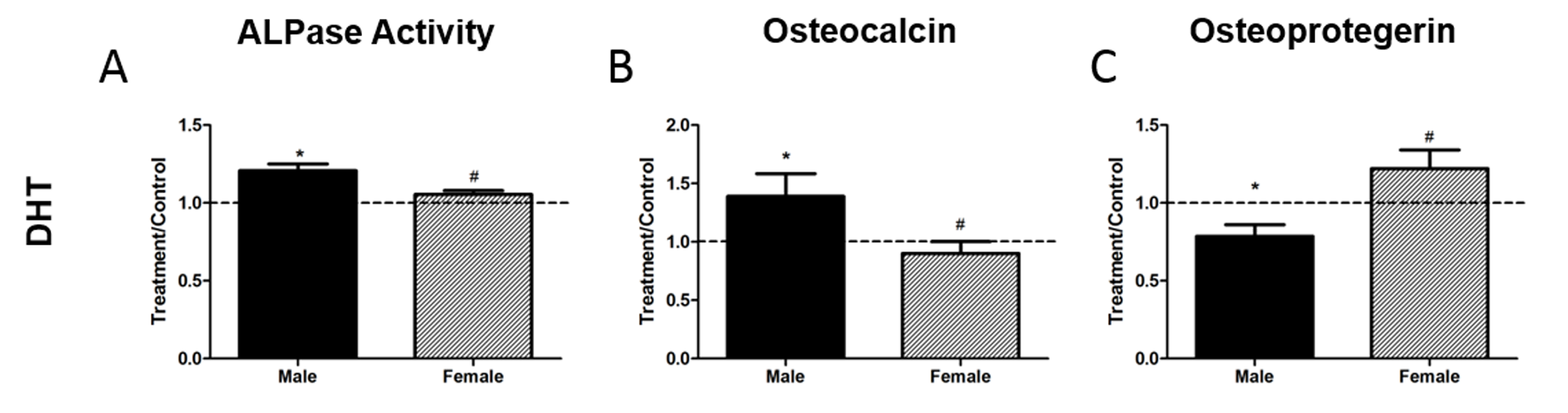

Supplement: Additional file 3: Figure S2. — Phenotypic characteristics of female and male primary osteoblasts isolated from osteoarthritic knees. First-passage osteoblasts were treated with 10−8 M DHT. Alkaline phosphatase-specific activity (A) was measured in whole cell lysates. Protein levels of osteocalcin (B) and osteoprotegerin (C) were measured in conditioned media. Data show treatment compared with vehicle control ratios of the responses of 6 male and 6 female patients. The dashed line represents the vehicle control (dashed line = 1). * p < 0.05 vs. control; † p < 0.05 vs. male. (TIF 1003 kb) [file 13293_2016_80_MOESM3_ESM.tif]
